# Ultrasound-targeted microbubble destruction promotes myocardial angiogenesis and functional improvements in rat model of diabetic cardiomyopathy

**DOI:** 10.1186/s12872-020-01815-4

**Published:** 2021-01-07

**Authors:** Xijun Zhang, Xinqiao Tian, Peng Li, Haohui Zhu, Nanqian Zhou, Zhixin Fang, Yuping Yang, Yun Jing, Jianjun Yuan

**Affiliations:** 1 Department of Ultrasonography, Zhengzhou University People’s Hospital, Henan Provincial People’s Hospital, Henan University People’s Hospital, NO. 7 Weiwu Road, Zhengzhou, 450003 Henan China; 2 School of Pharmacy, Xinxiang Medical University, Xinxiang, 453002 Henan China

**Keywords:** Diabetic cardiomyopathy, Ultrasound microbubble contrast, Angiogenesis, Animal model

## Abstract

**Background:**

Microvascular insufficiency plays an important role in the development of diabetic cardiomyopathy (DCM), therapeutic angiogenesis has been mainly used for the treatment of ischemic diseases. This study sought to verify the preclinical performance of SonoVue microbubbles (MB) combined ultrasound (US) treatment on myocardial angiogenesis in the rat model of DCM and investigate the optimal ultrasonic parameters.

**Methods:**

The male Sprague–Dawley (SD) rats were induced DCM by streptozotocin through intraperitoneal injecting and fed with high-fat diet. After the DCM model was established, the rats were divided into the normal group, DCM model group, and US + MB group, while the US + MB group was divided into four subsets according to different pulse lengths (PL) (8 cycles;18 cycle;26 cycle; 36 cycle). After all interventions, all rats underwent conventional echocardiography to examine the cardiac function. The rats were sacrificed and myocardial tissue was examined by histology and morphometry evaluations to detect the myocardial protective effect of SonoVue MBs using US techniques.

**Results:**

From morphologic observation and echocardiography, the DCM rats had a series of structural abnormalities of cardiac myocardium compared to the normal rats. The US-MB groups exerted cardioprotective effect in DCM rats, improved reparative neovascularization and increased cardiac perfusion, while the 26 cycle group showed significant therapeutic effects on the cardiac functions in DCM rats.

**Conclusion:**

This strategy using SonoVue MB and US can improve the efficacy of angiogenesis, even reverse the progress of cardiac dysfunction and pathological abnormalities, especially using the 26 cycle parameters. Under further study, this combined strategy might provide a novel approach for early intervention of DCM in diabetic patients.

## Background

Diabetic cardiomyopathy (DCM), caused by diabetes, is a common cardiovascular complication independent of coronary artery disease and hypertension [1]. It has a set of changes in the myocardial structure and function and has been receiving extensive attention due to the associated morbidity and fatality rates [2, 3]. However, DCM is often neglected in the clinical process because of no special symptoms in the early stage and lack of specialized treatment strategies [3–6]. Therefore, reveal the subtle changes of cardiac mechanics are important for patient management and intervention.

Microvascular insufficiency plays an important role in the development of DCM [4]. Accumulating evidence shows that the impaired angiogenic response to chronic ischemia may lead to reduced perfusion of myocardium, which finally contributes to interstitial fibrosis, oxidative tissue injury, and heart failure [7, 8]. Therefore, promoting cardiac angiogenesis and improving microcirculation function has become a potential therapeutic target for the treatment of DCM [8–11].

Recently, therapeutic angiogenesis has been mainly used for the treatment of ischemic diseases [9, 10]. However, previous studies have reported the delivery approach of proteins and genes, including intracoronary, intrapericardial or myocardial administration, are inefficient and high risk that prevent its clinical applications. At the moment, ultrasound-targeted microbubble destruction (UTMD) has been developed as a noninvasive and target specific method in angiogenesis therapy of cardiovascular disease [5, 12, 13].

UTMD technique has acquired much attention as an effective method to deliver basic fibroblast growth factor (bFGF) to the heart, and the resulting growth factor therapy has obviously reverse cardiac dysfunction and even the structure of damaged cardiac tissues [12]. However, bFGF has a potential tumorigenic ability, so the lack of safety limits its application in vivo.

**Fig. 1:**
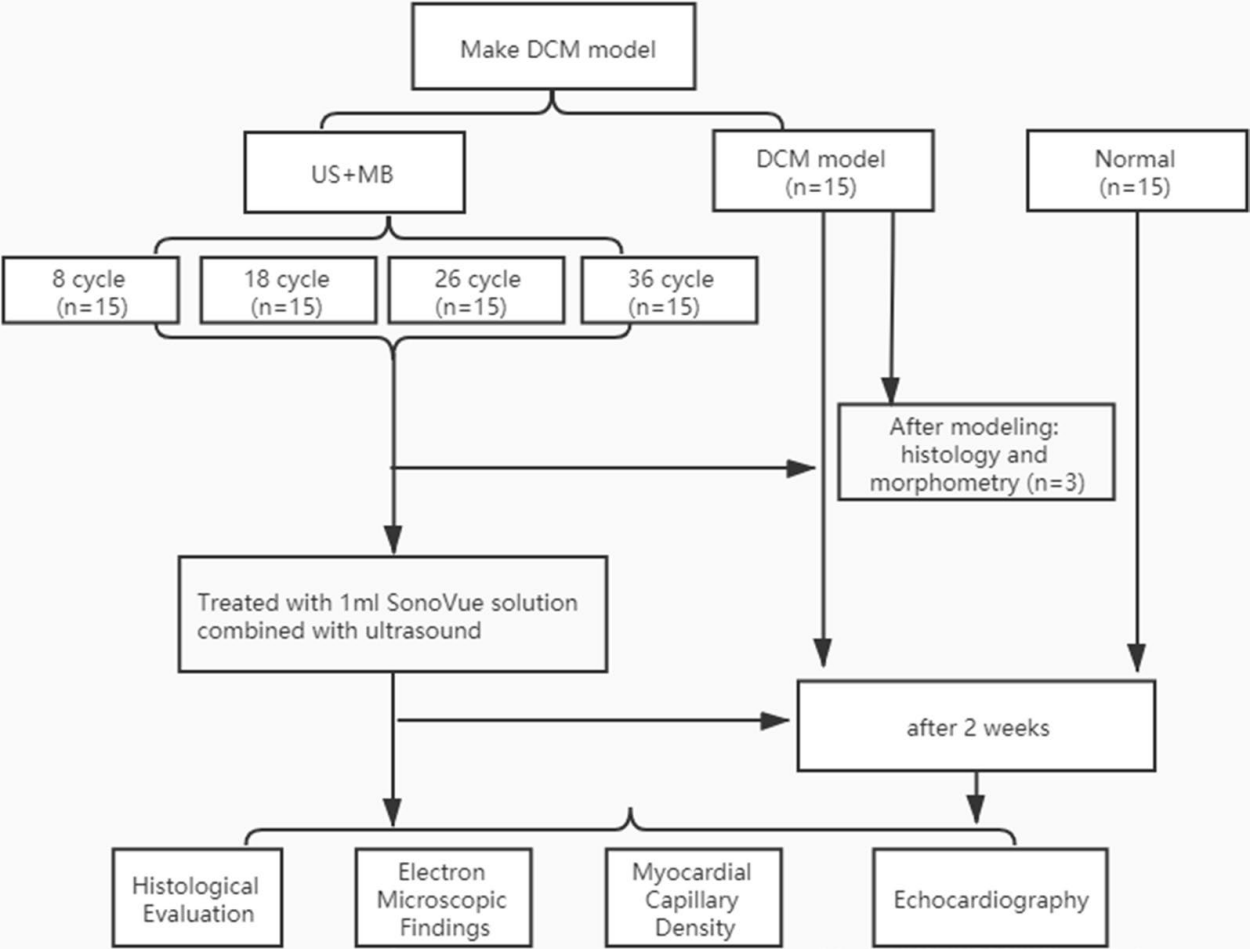
Flow chart of study design

As an ultrasound contrast agent, SonoVue (Bracco International BV, Amsterdam, Netherlands) showed high safety, good contrast-enhanced images in clinical use [14–16]. The present study aims to investigate the effects of SonoVue/UTMD combined treatment on myocardial angiogenesis and inspect the optimal ultrasonic parameters in a rat model of DCM. The experimental details were shown in Fig. 1.

## Methods

### Microbubble preparation

We used an ultrasound contrast agent, Sonovue (Bracco International BV, Amsterdam, Netherlands) as microbubbles (MB). It was dissolved in 5 ml physiological saline to make a SonoVue colloid dispersion, which contained 60.7 mg SF6 and 25 mg lyophilized powder. The MB concentration in the solution was measured by cell counting microscopic method. The MB concentration in the suspension was about 2 × 10^8^ bubble/mL with an average diameter of 3.4 μm. The diluted solution of microbubble suspension (1 mL microbubble suspension in 19 mL normal saline, which was diluted by 400 fold) was used in therapy. The therapeutic dose was about 1 mL/ piece, and the continuous infusion lasted for 20 min.

### DCM animal models

All animal experiments in this study were approved by the Animal Care and Use Committee of Xinxiang Medical College and conducted by the “Guide for the Care and Use of Laboratory Animals” from the National Institutes of Health. Sprague- Dawley (SD) rats were widely used as an animal model for studying diabetes mellitus (DM), which were suitable for the evaluation of cardiac function [5, 6, 12, 13, 17]. Male rats (7–8 weeks, 160–180 g) were provided by the Experimental Animal Center of Zhengzhou University. Diabetes was induced by a single intraperitoneal injection of streptozotocin (STZ, Sigma Corporation, USA) at 65 mg/kg after 12 h of fasting, which had a similar progression of human’s DM. The normal control rats were administered with the same volume of normal saline. After one week, the DM rats were provided with a fresh high fat-diet (basal diet added with 18% W/W fat oil and 20% W/W glucose) for 8 consecutive weeks, while the control group was fed with a normal diet. The fasting tail blood glucose was measured on the 3rd day, 7th day, and 9th week after the STZ injection. The rats with blood glucose over 16.6 mmol/L were selected as DM rats using in the following study [17–19].

### Groups and treatments of animals

**Fig. 2:**
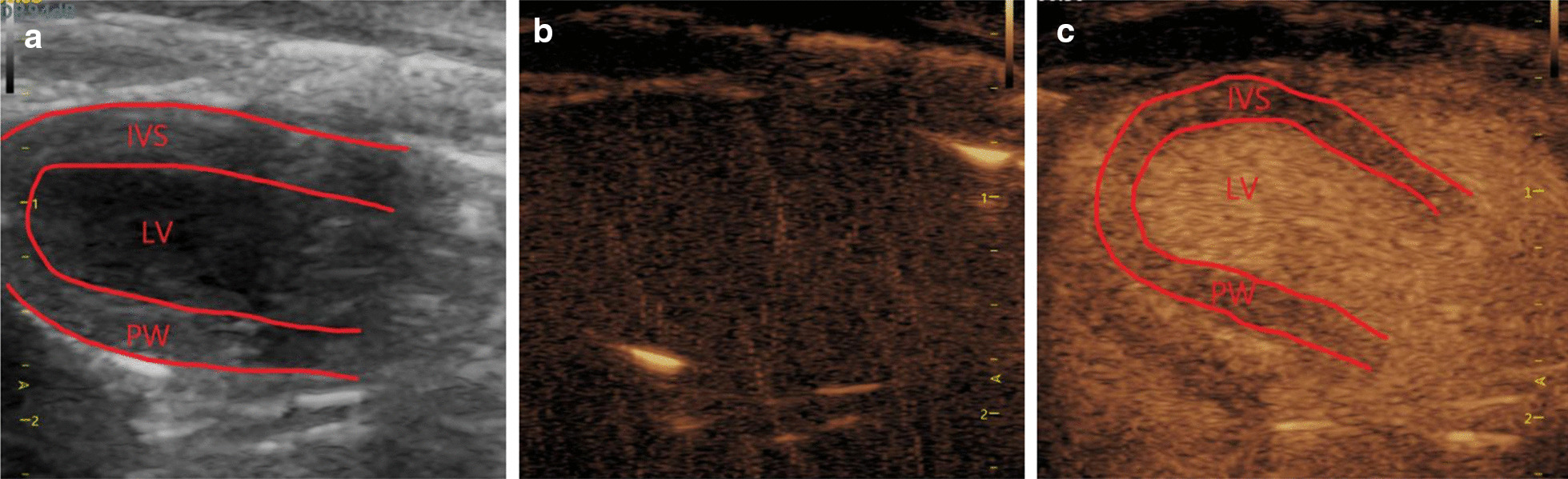
Contrast ultrasonography of SonoVue MB in rat heart. **a** 2D echocardiograms, **b** ultrasound images before the filling of MB. **c** left ventricular was filled with MB

The rats were anesthetized using an intraperitoneal injection of 30 mg/kg sodium pentobarbital. The thoracic region was carefully shaved and placed supine on a warm procedure board. The VINNO 70 color Doppler diagnosis system (VINNO, Suzhou, China) combined with VFLASH (a software that can manipulate microbubble cavitation) was utilized to generate the UTMD effect, which was performed using a 14 MHz linear-array transducer. The traditional echocardiographic measurements were obtained by M-mode echocardiography at left ventricular long-axis views (Fig. 2a), including the left ventricular internal dimension at end-diastole (LVIDd), the thickness of interventricular septum at end-diastolic (IVS), left ventricular fraction shortening (LVFS) and left ventricular ejection fraction (EF).

After satisfactory images were obtained (Fig. 2b), a dosage of 1 ml diluted SonoVue solution was administered into the tail vein continuously over 20 min (Fig. 2c). At the same time, the VFLASH mode of the scanner was switched on for UTMD (frequency: 4 MHz, pulse frequency: 20 Hz, exposure time = 1.2 s, US exposure duration per time = 2 s) and the heart was in the region of interest (ROI) during the experiment.

The animals were randomly divided into three groups: (1) Normal group (n = 15): non-diabetic rats were injected 1 ml saline solution; (2) DCM model group (n = 15): DCM rats were injected 1 ml saline solution. (3) US + MB group: DCM rats were treated with 1 ml SonoVue solution combined with ultrasound (US). The third group was divided into four subsets according to different pulse lengths (PL) (8 cycles; 18 cycle; 26 cycle; 36 cycle) (n = 15 for each group). For all animals, the second treatments were processed after one week of the first treatments.

### Histology and morphometry

All specimens were anesthetized using an intraperitoneal injection of 30 mg/kg sodium pentobarbital, then euthanized by thoracotomy and heart removal. Three rats from the control group and DCM group were euthanized after modeling to evaluate the pathological changes of the pancreas and myocardium. After two weeks of all the intervention, the rats were sacrificed, then the left ventricular myocardium was fixed with 10% paraformaldehyde and embedded in paraffin. Sections of papillary muscle (about 5 mm thick) were stained with hematoxylin and eosin (HE) to observe the morphology.

CD31 immunohistochemistry was used to identify the myocardial capillary density (MCD). A total of 20 high power fields (400×) were randomly selected and the number of brown-stained capillaries was counted and averaged as MCD [20].

### Statistical analysis

All the data was graphed and analyzed in Microsoft Excel (Microsoft Corporation, Redmond, WA, USA) and SPSS statistical software (13.0 SPSS Inc., Chicago, IL, USA). The data were expressed as the mean ± SD. Statistical comparisons were determined using one-way ANOVA and LSD-t test. Dunnett’s T3 test was performed on the variance, and significance levels were defined at *P* < 0.05.

## Results

### General condition of experimental rats

After two weeks of all intervention, the final number of rats completed the study in each group were: 12 in the DCM model group, 12 in the 8 cycle group, 14 in the 18 cycle group, 13 in the 26 cycle group, 12 in the 36 cycle group, and 14 in the normal control group.

**Fig. 3:**
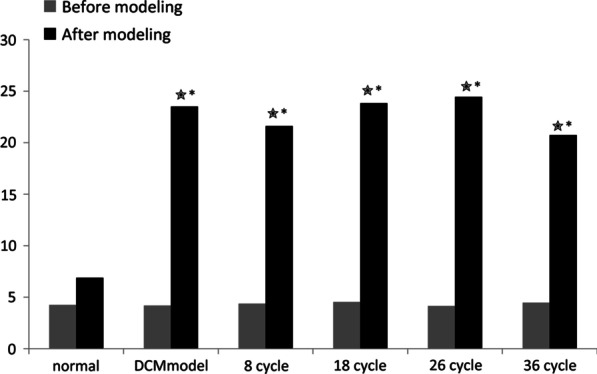
Blood glucose levels of rats after modeling. Comparing with normal group, blood glucose in model group and treatment group increased significantly (*, *P* < 0.05). After modeling, blood glucose levels increased significantly (✭, *P* < 0.05)

For blood glucose analysis, rats fasted overnight and blood samples were collected. Serum was separated and levels of glucose were measured. The glucose values after modeling were exceeding 16.6 mmol/L. There was a significant increase in blood glucose levels after modeling compared to those before modeling with a *P-*value < 0.05 (Fig. 3) for all groups except the normal control group. A distinct difference was observed in blood glucose levels between DCM rats and the normal control groups (*P* < 0.05, Fig. 3).

### Histological evaluation

**Fig. 4:**
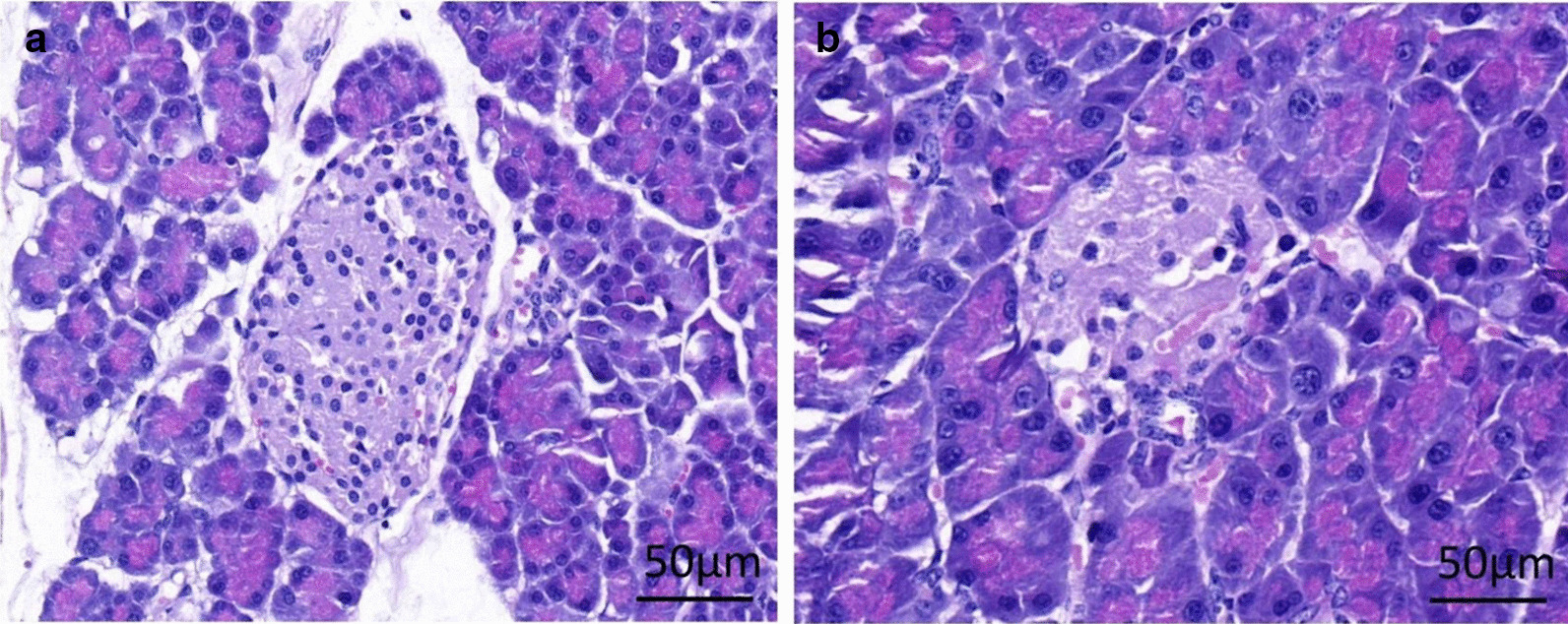
The histological changes of the pancreas after modeling. **a** The normal group; **b** the DCM model group

After modeling, as shown in Fig. 4, the pancreatic tissue structure was complete and regular, the islet cells were compact and uniform, and the cytoplasm was abundant without vacuolation (Fig. 4a). Therefore, the pancreatic tissue of the DCM rats was seriously damaged, and the islet cells showed degenerative atrophy, only a few degenerative islet cells were sparsely distributed (Fig. 4b).

**Fig. 5:**
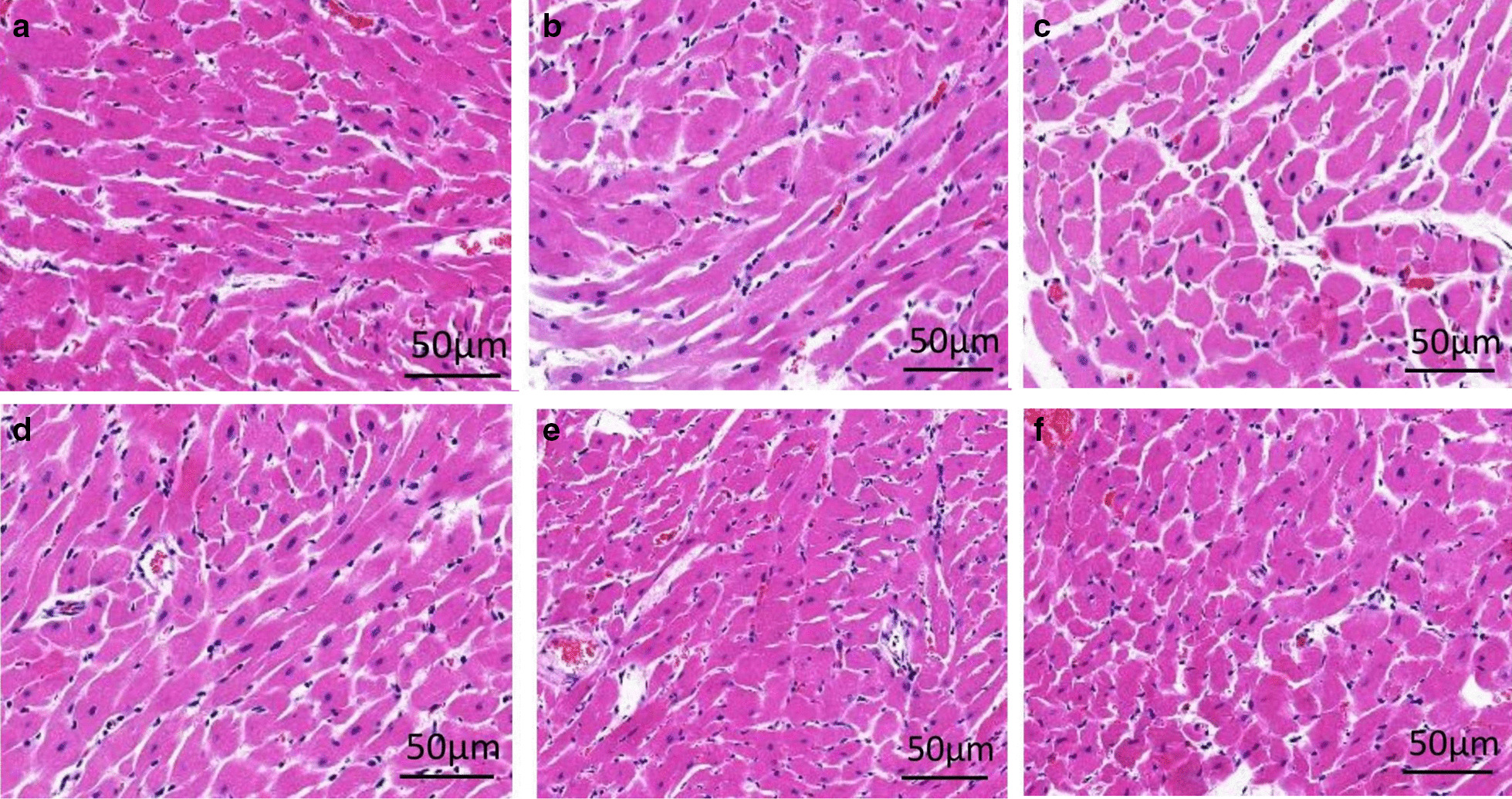
The histological changes of the heart after intervention. **a** Normal group, **b** DCM model group, **c** 8 cycles group, **d** 18 cycle group, **e** 26 cycle group, **f** 36 cycle group

After two weeks of all interventions, as shown in Fig. 5, the cardiomyocytes were intact with a normal clear structure, and the nucleus was oval and located in the center of the normal group (Fig. 5a), while a large number of hypertrophic and necrotic cardiomyocytes were observed in the DCM model group, which were distributed in an altered and disorganized arrangement with obvious vacuolization (Fig. 5b). Nuclear pyknosis and myocardial fibrosis increased significantly in the DCM model group. The DCM rats had a set of structural abnormalities in the heart, and therefore, the US + MB treatment groups (Fig. 5d–f) showed decreased hypertrophy, fibrosis and cardiomyocyte lesions.

### Electron microscopic findings

**Fig. 6:**
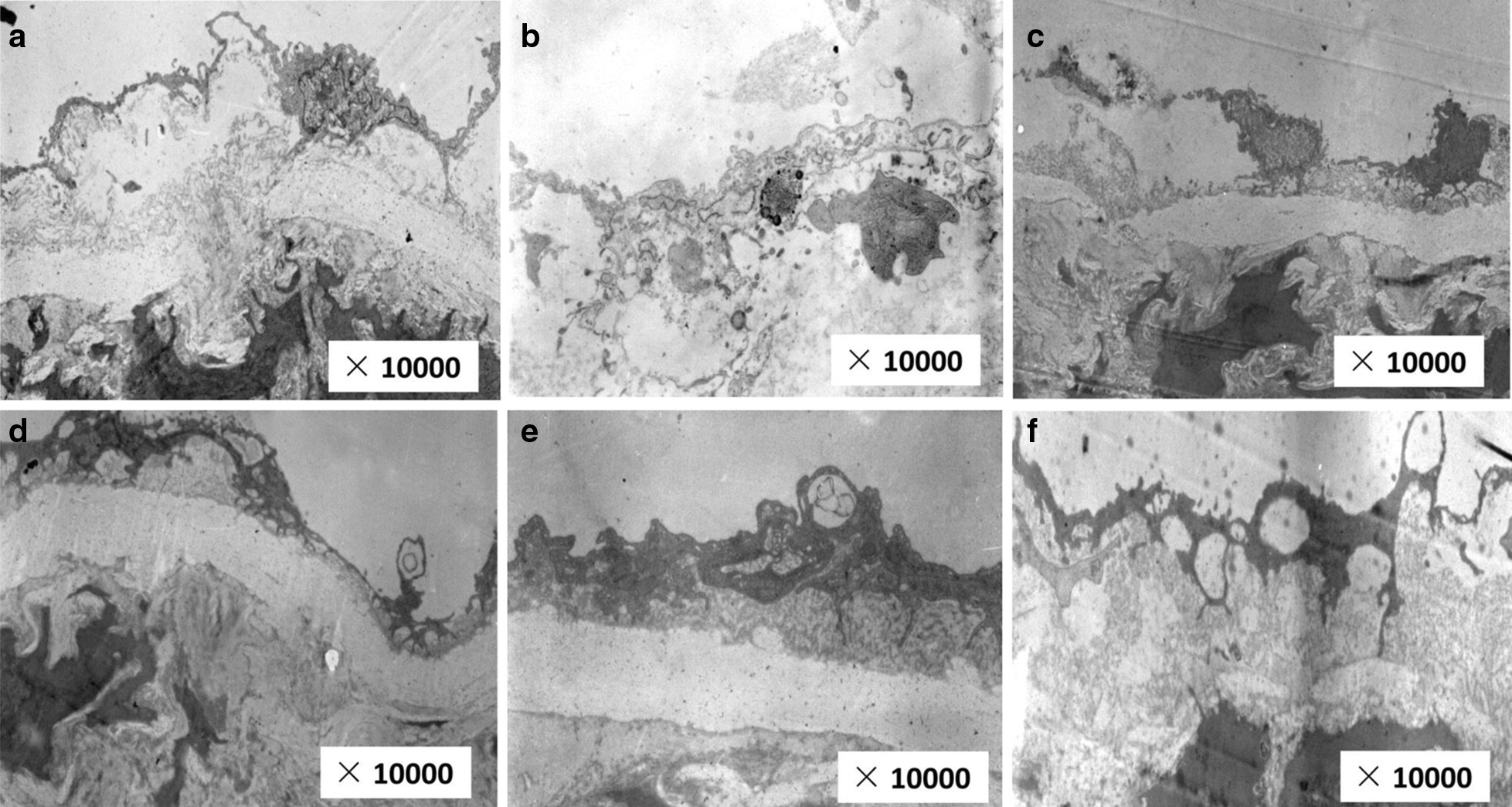
Representative pictures of electron micrographs (10,000 ×) of cardiac microvascular from the rats of each group. **a** Normal group, **b** DCM model group, **c** 8 cycles group, **d** 18 cycle group, **e** 26 cycle group, **f** 36 cycle group

To evaluate the changes of microvascular under different intervention conditions, the ultrastructural changes of the cardiac microvascular were examined under electron microscope. As shown in Fig. 6, in the normal group, the surface of cardiac microvascular was smooth, there was no obvious convexity, and the end structure of blood vessels was intact, while the surface of cardiac microvascular in the model control group was more burred and continuality was missing, the endothelial cell membrane was incomplete, and the heterochromatin of the inner nucleus increased and agglomerated, which indicated the pathological state of these microvessels. In the 36 cycle group, myocardial arterioles were not smooth, continuity was missing, endothelial cell membrane was incomplete, nuclear heterochromatin increased and agglomerated, showing a state of injury. In the 8 cycle group, 18 cycle group, and 26 cycle group, the surface of cardiac microvascular were smooth and continuous, the endothelial cell membrane was intact, and the cytoplasmic mitochondria were angiogenesis, while the myocardial samples in the 26 cycle group showed the relatively well-integrated ultrastructures compared with other US-MB treatment and DCM group.

### Echocardiography evaluation

The traditional echocardiographic measurements including LVIDd, IVS, LVEF, LVFS and HR were acquired to estimate the improvement of global myocardial contractile function after two weeks of all intervention (Table 1). There was statistically significant difference in LVEF, LVFS and HR in the DCM model group and normal control group (*P* < 0.05). A distinct improvement was observed in DCM model rats in the levels of the LVEF, LVFS and HR with a *P* value < 0.05 after UTMD intervention. The LVEF and LVFS in the US + MB subgroup were significantly increased compared with the DCM model group (all *P* < 0.05). Among all the US + MB groups, the 26 cycle group showed the highest LVEF and LVFS after treatment, and had no statistically significance when compared to the normal group (*P* > 0.05). There was no statistical significant difference in HR in the 8 cycle group and DCM group after the intervention, but a distinct difference was observed between the DCM group and other US-MB groups in HR with a *P* value < 0.05. There was a significant increase in the level of HR in the US-MB group but they still did not reach the levels of the normal control rats. There was no statistically significant difference in IVS and LVIDd in UTMD group and DCM model group with a *P* value > 0.05 after the intervention.

**Table 1:** Basic Echocardiographic data of the rats in each group ($\overline{x} \pm s$)

Characteristics	Normal	DCM model	US + MB Subgroups			
			8 cycle	18 cycle	26 cycle	36 cycle
LVIDd (mm)	7.42 ± 0.21	7.44 ± 0.41	7.36 ± 0.48	7.23 ± 0.69	7.41 ± 0.78	7.61 ± 0.69
IVS (mm)	1.61 ± 0.16	1.54 ± 0.19	1.51 ± 0.14	1.62 ± 0.17	1.55 ± 0.18	1.59 ± 0.18
LVEF (%)	73.51 ± 4.44#	57.76 ± 6.03*	64.48 ± 5.12*#	65.59 ± 6.27*^#^	68.97 ± 4.84^#^	63.49 ± 5.34*#
LVFS (%)	35.06 ± 3.15#	27.70 ± 3.24*	29.94 ± 3.55*#	31.88 ± 4.28*^#^	34.01 ± 3.97^#^	30.78 ± 3.87*^#^
HR	378.30 ± 14.44^#^	288.78 ± 15.82*	301.67 ± 16.68*	321.21 ± 29.58*^#^	331.13 ± 20.95*^#^	307.42 ± 23.81*^#^

LVIDd, left ventricular internal dimensions; IVS, interventricular septum dimensions; LVEF, left ventricular ejection fraction; LVFS, percentage of left ventricular fractional shortening; HR, heart rate

*P < 0.05, VS normal group; #*P* < 0.05, VS model group

### Myocardial capillary density

As shown in Finger 7, the DCM group demonstrated a gradual decrease in the MCD of myocardial tissue when compared with that in the control group (*P* < 0.05). Moreover, each US-MBs intervention group showed significant increase in MCD than that in the DCM control group, but the increase of MCD in 8 cycle group had no statistical significance when compared to the DCM control group (*P* > 0.05), while the other US-MB groups were significantly higher than that in the DCM model group (*P* < 0.05). Among all the US-MB groups, the level of MCD in the 26 cycle group showed no statistically significant difference compared to the normal group (*P* > 0.05).

## Discussion

Microcirculatory dysfunction is believed to play an important role in DCM. Accumulating evidence has demonstrated a progressive reduction of the microvasculature and an impaired angiogenic response to chronic ischemia with the development of diabetes [8]. Recently, low-intensity ultrasound in combination with microbubbles has been shown to stimulate endogenous vascular growth factor, inducing angiogenesis, used in ischemic diseases [5, 21, 22]. As gas-filled colloidal materials, SnonVue MBs consisted of an inert gas core and shell composition of phospholipid, polymer with a typical mean diameter of 2.5 μm. It is a biocompatible and biodegradable material that has been extensively utilized in clinics with sufficient bio-safety. In this study, we evaluated the in vivo therapeutic effects of SonoVue /UTMD combined treatment in a rat model of DCM and explored optimal parameters of ultrasound devices in different group settings.

In this study, both the blood glucose levels (Fig. 3) and histological changes of the pancreas and hearts (Figs. 4, 5) proved that the DCM rats were successfully induced after modeling. Then the rats were divided into six groups according to the different interventions. After all treatments, as shown in Table 1, a significant decrease of the LVEF, LVFS and HR were observed in the DM model group compared with the normal control group (*P* < 0.05). Consistent with previous report [13, 23], the diabetic rats left untreated for 12 weeks were characterized by a declined systolic myocardial performance and had a set of structural abnormalities in the heart compared to the control group. Therefore, it is very important to prevent the occurrence of DCM by intervening before the obvious pathological changes in myocardium in DCM rats.

**Fig. 7:**
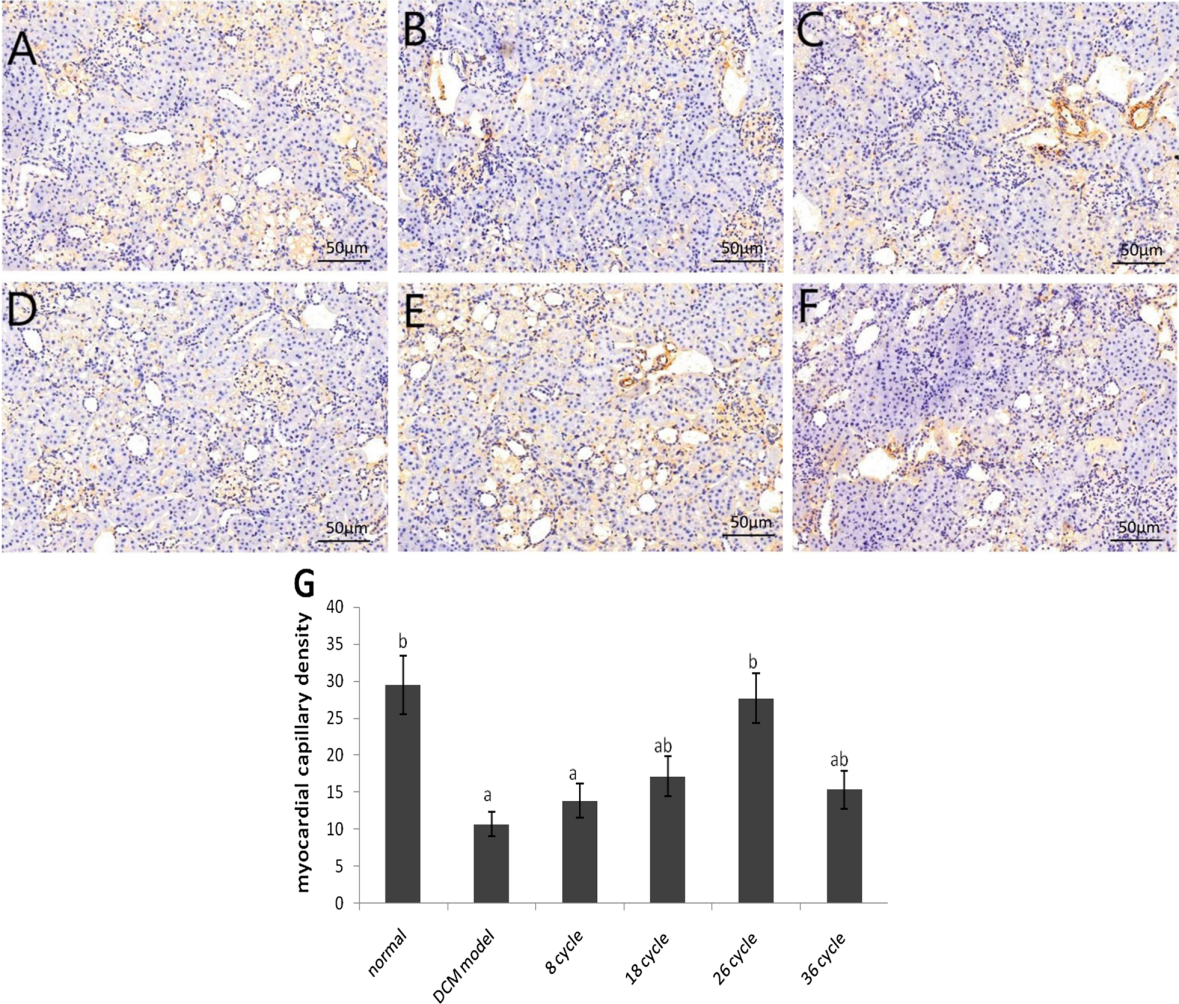
Representatives of CD31 immunohistochemical staining of MCD and the semi-quantitative analysis for all groups (400×). **A** normal group, **B** DCM model group, **C** 8 cycles group, **D** 18 cycle group, **E** 26 cycle group, **F** 36 cycle group, **G** Statistical histogram. **a** *P* < 0.05 versus the normal control group, **b** *P* < 0.05 versus the DM model group

All of the echocardiography evaluation criteria (Table 1) showed that the levels of LVEF and LVFS were significantly improved, along with a good change of HR between the US-MBs treatment group and the DM model group, while LVEF, LVFS and HR in the 26 cycle group were significantly higher than those in the other US-MB intervention groups. The results indicated that UTMD combined with Sonovue MBs could significantly restore the cardiac functions in DCM animals, especially when the US pulse length parameter is 26 cycles. The histochemical staining data presented in Figs. 5, 6 and 7 showed signs of moderate structural recovery in the US + MB treatment group compared to the DCM model group, further suggested that the improved functions were the results of structural remodeling of the cardiac tissues and thus may bring longer lasting therapeutic benefit.

US-MBs intervention could increase microvascular density, reverse the ultrastructures of myocardial microvascular injury, improve reparative neovascularization and increase cardiac perfusion. As the diabetes disease progresses, the microangiopathy exerts changes in the morphology and density of microvasculature [24]. As shown in Fig. 6, alterations in the surface of cardiac microvascular and destruction in endothelial cell membrane were observed in the myocardial samples by electron microscopy in DCM rats compared with the normal control ones. There were similar alterations in the ultrastructures of the surface of cardiac microvascular, as observed by electron microscope, in the group with UT-MBs treatment compared with the DCM group (Fig. 6). In our study, the CD 31 immunohistochemical assay (Fig. 7) also confirmed comparable therapeutic effect against DCM-related microangiopathy. The complex mechanism of angiogenesis using US-MBs treated in DCM model remains to be explored, it may be related to two factors. First of all, cavitation and mechanical effects of blasting could increase oxidative stress and induce local inflammatory factors, and then release vascular growth factors to activate angiogenesis [21]. Secondly, increasing the blood flow using the US-MBs treatment is most likely mediated by cavitation-related increases in shear and activation of endothelial nitric oxide synthase [25]. Huang et al. [26] suggested that the PI3K/Akt signal pathway might participate in modulating the activity of eNOS. Akt signaling protects against myocyte apoptosis induced by cardiac ischemia–reperfusion injury and DCM [27, 28]. The protective mechanism of US-MBs promoting angiogenesis may be associated with the activation of the PI3K/Akt signaling pathway by the up-regulation of expression of phosphorylated Akt protein to activate eNOS [27, 29].

Among various US-MBs treated groups, we found that different pulse length could achieve different intervention results. There were well-integrated ultrastructures in the myocardial samples in the 26 cycle group compared with other US-MBs treatment and DCM group, while it also demonstrated the highest effectiveness in increasing density of microvasculature. Furthermore, the routine echocardiography (Table 1) showed that the 26 cycle group significantly restored the cardiac functions in DCM rats. Therefore, this method can be used as an effective strategy to prevent the deterioration of cardiac function in DCM rats. This pre-clinical study provides us with great hope for early intervention of DCM in diabetic patients in the future. We predict that once the delivery system is optimized, this combined strategy might be used as an effective option for preventing DCM development.

## Conclusion

In conclusion, while using non-viral vectors SnonVue MBs combined with UTMD technique can significantly improve or even reverse cardiac dysfunction and pathological abnormalities, especially using the 26 cycle parameters. Under further study, this combined strategy might be a promising option for early intervention of DCM in diabetic patients.

## Data Availability

The datasets used or analysed during the current study are available from the corresponding author on reasonable request.

## Abbreviations

DM: Diabetes mellitus
DCM: Diabetic cardiomyopathy
UTMD: Ultrasound-targeted microbubble destruction
bFGF: Basic fibroblast growth factor
MBs: Microbubbles
MB: Microbubble
STZ: Streptozotocin
LVEF: Left ventricular ejection fraction
LVIDd: Left ventricular end-diastolic diameter
IVS: Interventricular septum dimensions
LVFS: Left ventricular fraction shortening
HE: Hematoxylin and eosin
MCD: Myocardial capillary density
HR: Heart rate
